# Modelling the effects of social distancing, antiviral therapy, and booster shots on mitigating Omicron spread

**DOI:** 10.1038/s41598-023-34121-y

**Published:** 2023-04-27

**Authors:** Jongmin Lee, Renier Mendoza, Victoria May P. Mendoza, Jacob Lee, Yubin Seo, Eunok Jung

**Affiliations:** 1 Department of Mathematics, Konkuk University, Seoul, 05029 South Korea; 2 Institute of Mathematics, University of the Philippines Diliman, Quezon City, 1101 Philippines; 3 Division of Infectious Disease, Department of Internal Medicine, Kangnam Sacred Heart Hospital, Hallym University College of Medicine, Seoul, 07441 South Korea

**Keywords:** Computational models, Viral infection, Applied mathematics

## Abstract

As the COVID-19 situation changes because of emerging variants and updated vaccines, an elaborate mathematical model is essential in crafting proactive and effective control strategies. We propose a COVID-19 mathematical model considering variants, booster shots, waning, and antiviral drugs. We quantify the effects of social distancing in the Republic of Korea by estimating the reduction in transmission induced by government policies from February 26, 2021 to February 3, 2022. Simulations show that the next epidemic peak can be estimated by investigating the effects of waning immunity. This research emphasizes that booster vaccination should be administered right before the next epidemic wave, which follows the increasing waned population. Policymakers are recommended to monitor the waning population immunity using mathematical models or other predictive methods. Moreover, our simulations considering a new variant’s transmissibility, severity, and vaccine evasion suggest intervention measures that can reduce the severity of COVID-19.

## Introduction

Designated by the World Health Organization as a variant of concern on November 26, 2021, Omicron has become the dominant variant of COVID-19^1^. Studies have shown that Omicron causes less severe infections. However, Omicron is more transmissible^2^ and has a greater ability to evade immunity than Delta^3^. Moreover, Omicron has a higher chance of reinfection compared to previous variants^4,5^.

Vaccine effectiveness and immunity induced by vaccines or a prior infection vary depending on SARS-CoV-2 variants and wane over time^3,5–7^. Despite these, vaccination is still an important measure in protecting the population. Booster vaccines have been rolled out in many countries with priority given to the most vulnerable groups^8^. Currently available vaccines remain effective against severe disease and death, can slow down transmission, and may minimize the emergence of new variants^9^. Furthermore, non-pharmaceutical interventions (NPIs), such as wearing masks and social distancing (SD), have been crucial in interrupting and delaying the transmission of Omicron^10^.

The Republic of Korea’s COVID-19 pandemic response before vaccination started had centered on SD and Testing/Tracing/Treatment (3T strategy)^11^. From February 26, 2021, vaccination rolled out with priority given to the elderly and healthcare workers, followed by the younger age groups. A four-tier SD scheme was adopted in July 2021 that outlined the number of people in a gathering, and operational guidelines of various facilities^12^. During this time, SD was maintained at Level 2 (SD2) but was raised to Level 4 (SD4) on July 12, 2021, when the fourth wave prompted by the Delta variant began^13^. By October 2021, about 75% of the population had been fully vaccinated, and a decline in cases was observed. On November 1, 2021, the government implemented an eased SD level as part of its ‘gradual recovery to a new normal’ (GR) policy^14^. A sharp rise in daily cases of COVID-19 was seen towards the end of November until early December 2021 due to the eased SD and dominance of the Delta variant, which was detected in 96% of tested samples on December 2021^1^. Moreover, breakthrough infections (BTI) comprised 58.2% of the cases according to the data reported on January 17, 2022^1^. To maintain a high population-level immunity, more than 100,000 doses of booster shots have been administered per day since November 2021. The duration of getting a booster shot from the second dose had also been shortened from six to three months. Cases with the Omicron variant were first reported in Korea on December 1, 2021. On January 15, 2022, the proportion of infections with Omicron was around 26.7%^1^. A stronger SD policy called suspended GR (SGR) had been reinstated on December 18, 2021^15^. From the start of the SGR policy until February 3, 2022, the average daily confirmed cases and severe patients were 27,442 and 274, respectively.

On January 14, 2022, the COVID-19 antiviral drug Paxlovid was rolled out in Korea, the first Asian country to do so after authorizing its emergency use in December 2021^16^. Clinical trials show that Paxlovid has 89% effectiveness against severity^17,18^. From February 3, 2022, cases soared to more than 22,000 per day, which corresponded to approximately 10% of the testing capacity. This prompted Korea to allow Rapid Antigen Testing done by a doctor as an official confirmation^19^. On March 16 and March 30, 2022, Korea recorded the highest number of daily confirmed cases and severe patients with 621,317 and 1315, respectively. From February 3 to November 30, 2022, booster vaccines have been administered to about 14 million^8^.

Mathematical models have been extensively used to understand the dynamics of COVID-19 in various countries^20^. They have also been utilized in proposing strategies to ease the effects of the pandemic^21^. Furthermore, vaccination roll-out strategies were designed using mathematical models^22^. Various models considering the Delta variant and Omicron variant have been presented^23^. Several papers suggested COVID-19 mathematical modeling related to machine learning and stochastic method^24–28^.

In this work, we propose a deterministic mathematical model that considers several relevant factors, including variants, vaccination, waning of natural immunity and vaccine effectiveness, booster shots, and antiviral therapy. We will estimate the parameters of the model using the available data. The objective of this study is to explore effective and timely vaccination strategies that can be applied to future waves of COVID-19, and taking into account the possibility of new variants. Our research findings enable policymakers to monitor the population’s immunity status and identify the best time to administer vaccines.

In the next section, we present the main results of the study, including the estimated parameters, calculations for waning and breakthrough infections, prevalence ratios of non-vaccinated and vaccinated groups, simulations on timing of booster vaccines, and projections in the event of a new COVID-19 variant. The discussion section highlights how the time-dependent parameter $\mu$ describes past situations and how it can be utilized to plan future strategies under different scenarios. This section also addresses the limitations of the study and offers avenues for future research. The conclusion section summarizes how the results of the study can be utilized to strategically plan PIs and NPIs to manage the COVID-19 pandemic. The Methods section provides details about the data collection process, mathematical model formulation, parameter estimation techniques, and experimental scenarios.

## Results

### Fitting results

**Figure 1:**
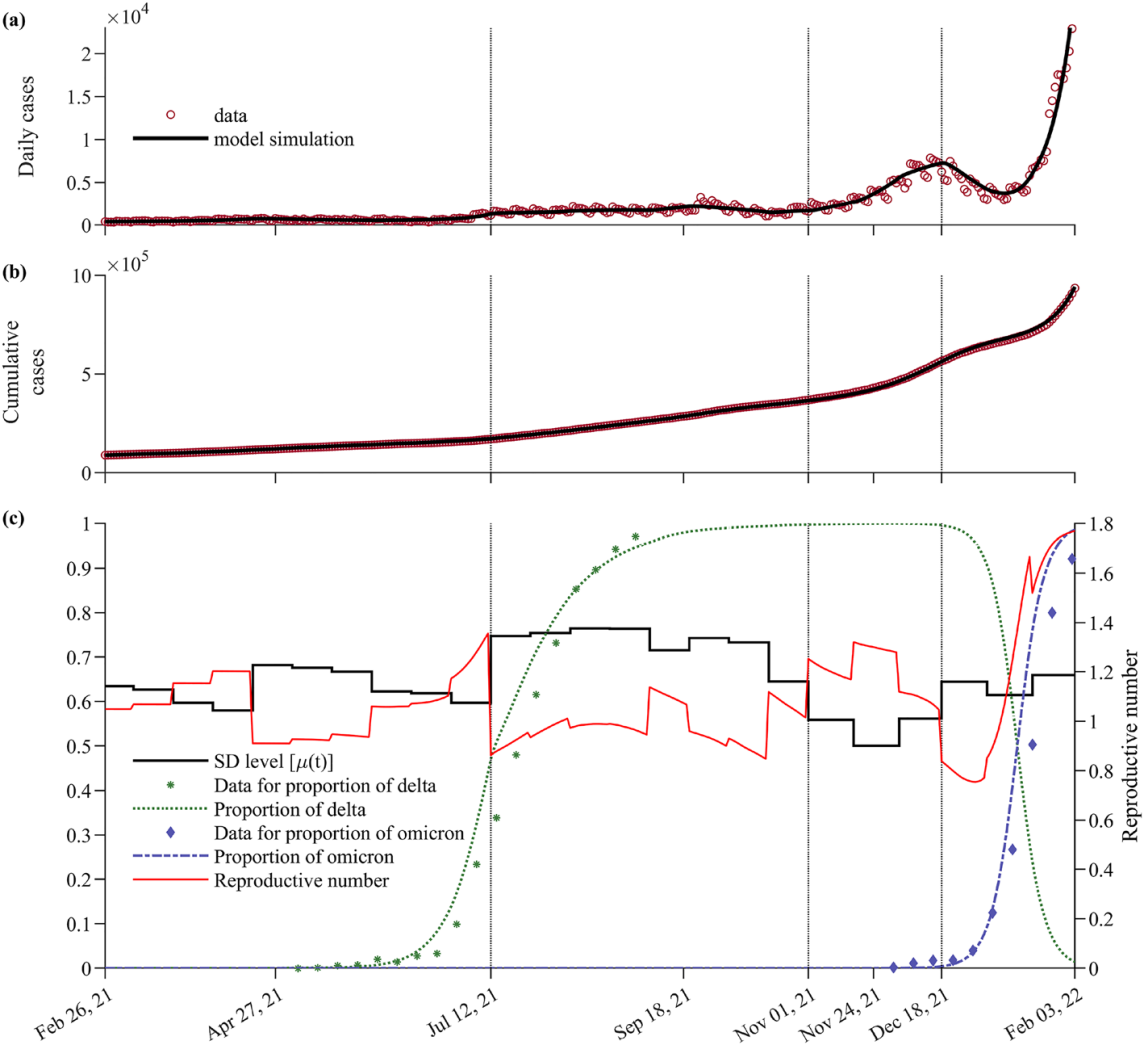
Parameter-fitting results for $\mu (t)$ from February 26, 2021 to February 3, 2022. The vertical dotted lines depict the transition between the different SD policies. Panels (**a,b**) show the best fit of the model to the daily and cumulative confirmed cases, respectively. The red circles represent the data on daily and cumulative cases. Panel (**c**) shows the estimated values of $\mu (t)$ (black curve), the effective reproductive number ${\mathscr {R}}(t)$ (red curve), and the proportion of Delta and Omicron variants among the confirmed cases (green and blue curves, respectively). The green and blue circles indicate the data on the proportion of Delta and Omicron infections among tested samples, respectively.

The estimation period is covered by four SD policies: SD2, SD4, GR, and SGR. The GR policy is an eased SD, aimed at gradual returning to normal life. We estimated $\mu (t)$, which quantifies the NPIs according to the SD policies of the government. Panels (a) and (b) of Fig. 1 show the best fit of the model to the daily and cumulative confirmed cases data (red circles), respectively. The vertical lines mark the changes in SD policy. The model captured the trends in the number of cases throughout the estimation period. It showed a rise in the daily cases during the GR phase, which peaked on December 18, 2021, and a decline shortly after SGR was implemented. Following the model’s trend, the cases were observed to increase sharply around February 2022.

The reproductive number ${\mathscr {R}}(t)$ (red curve) and the estimated $\mu (t)$ values (black curve) are shown in Panel (c). The symbols and dashed curves depict the data and model fit on the proportion of infections with the Delta and Omicron variants, respectively. During SD2, ${\mathscr {R}}(t)$ ranged between 0.9 and 1.2 except during the two weeks (including Chuseok, the second largest holiday in Korea) before the implementation of SD4, where ${\mathscr {R}}(t)$ jumped to 1.4. On the same two-week interval, the lowest value of $\mu (t)=0.58$ was estimated, and the proportion of cases with the Delta variant (green curve) increased steeply. From July 12 to October 31, 2021, Delta was the dominant variant of SARS-CoV-2 in Korea. The ${\mathscr {R}}(t)$ values ranged from 0.9 to 1.1. In the last 2 weeks before the policy was changed, the estimated $\mu (t)$ dropped to 0.64 and consequently, ${\mathscr {R}}(t)$ increased to 1.1. During the eased GR phase, ${\mathscr {R}}(t)$ remained greater than 1 and the mean value of $\mu (t)$ was 0.54. When an enhanced policy (SGR) was reinstated on December 18, 2021, the proportion of cases with the Omicron variant was only around 4%. In this period, the estimated $\mu (t)$ increased to 0.64, while ${\mathscr {R}}(t)$ ranged from 0.8 to 1.7. By February 3, 2022, the proportion of Omicron infections reached 98%. Supplementary A presents the mean value of the estimates $\mu (t)$ obtained on each SD phase.

### Comparison of the model simulations with the data from February to November 2022

**Figure 2:**
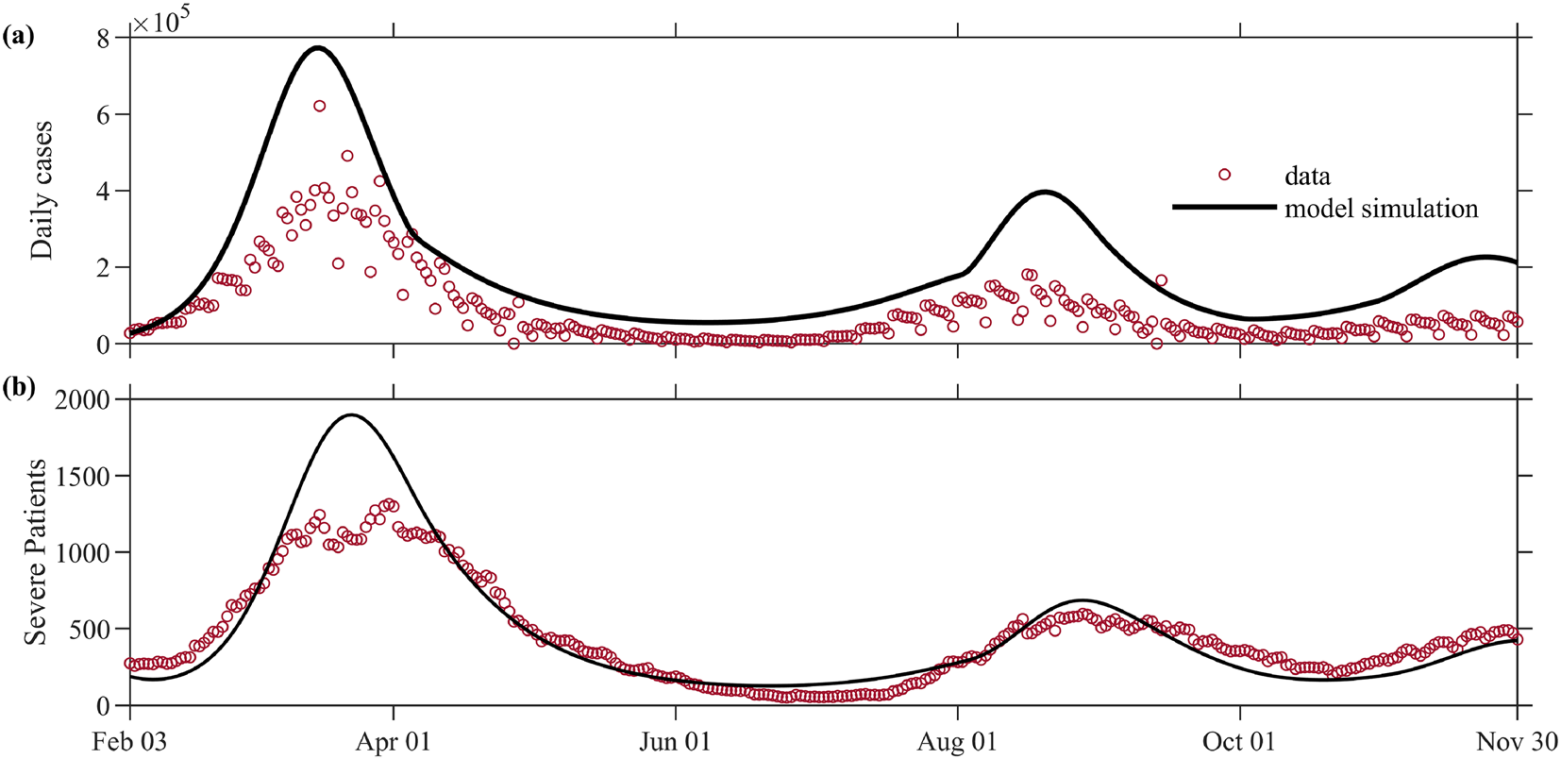
Comparison of the model simulations with the data from February 3 to November 30, 2022. Panel (**a,b**) show the daily confirmed cases and severe patients, respectively. The data are represented by the red circles.

Figure 2 shows the plots of the daily cases and severe patients using the model and vaccination data from February 3 to November 30, 2022. Here we assume decreasing $\mu$ values as described in the Methods section. The red dots represent the data for the daily cases and severe patients on the same period. Simulation results show the peaks of the daily incidence at 773,000 on March 16 and at 397,000 on August 20. The number of severe patients peaked at 1898 on March 23 and at 686 on August 28. In the first peak, we note the difference between the data and the model simulation results in the daily cases and severe patients.

**Figure 3:**
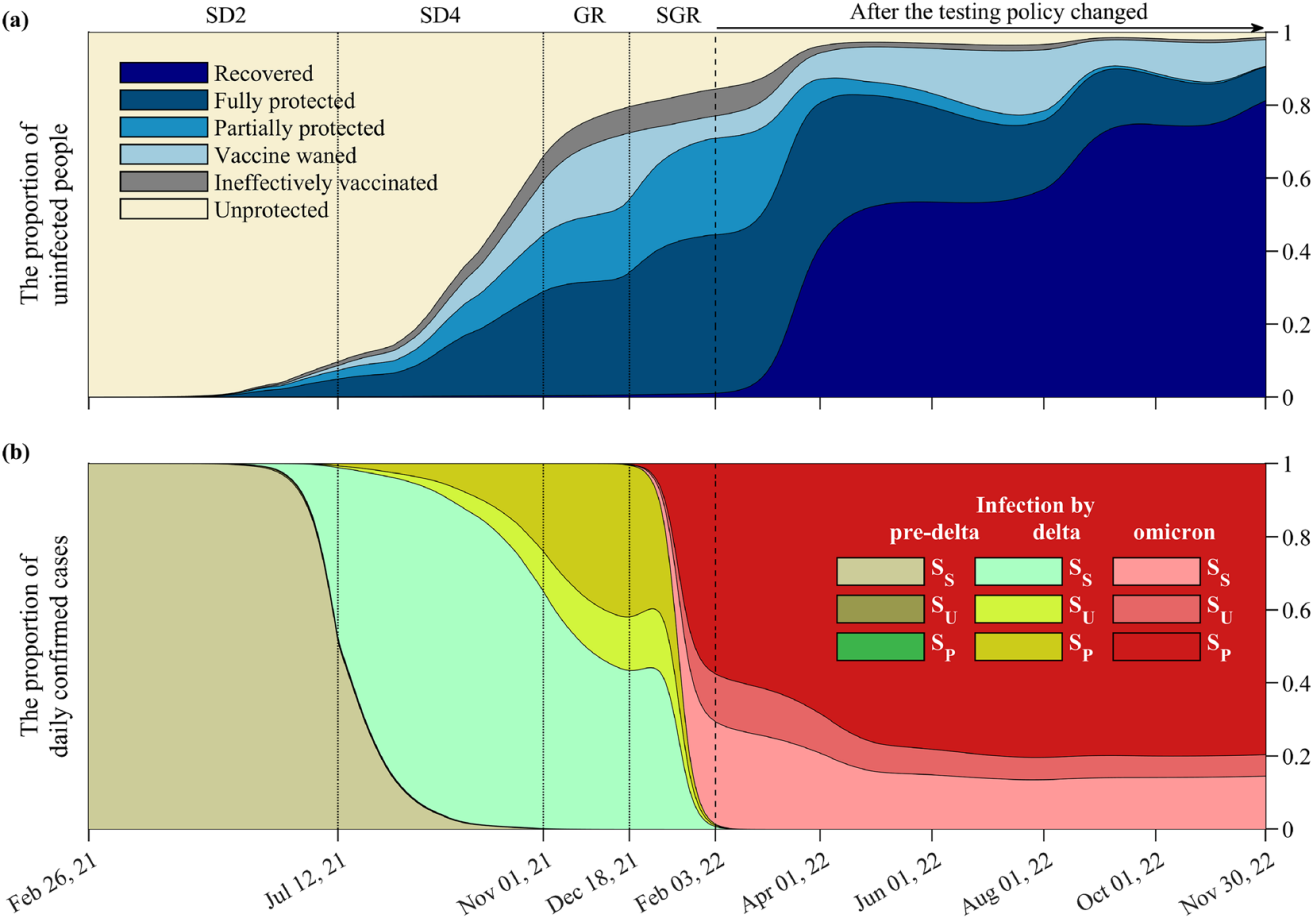
The proportion of (**a**) non-infected and (**b**) infected classes from February 26, 2021 to November 30, 2022. The black dotted lines depict the transition between the different SD policies. The solid black line on February 3, 2022 indicates the end of the estimation period and the start of the forecast period.

### Waning and breakthrough infections

Figure 3 shows the changes in the proportion of the non-infected (Panel (a)) and infected (Panel (b)) classes as more people were vaccinated and different SARS-CoV-2 variants arrived in Korea. The vertical line on February 3, 2022 marks the start of the prediction period, when the testing policy was changed. From July 12 to November 1, 2021, the proportion of vaccinated individuals (fully and partially vaccinated, waned, and ineffectively vaccinated) increased considerably from around 10 to 65%. This is consistent with the data on vaccine coverage (11.8% to 75%^8^). In the subsequent GR phase, the waned class (light blue) constituted more than one-fifth of the non-infected population. Since the administration of booster shots started in October 2021, the proportion of fully-protected individuals in the SGR phase increased again. In the prediction, simulations show that the proportion of the fully protected and recovered classes oscillates between 70 and 90%. Towards the end of November 2022, the model shows less oscillations and the proportion of the recovered class increased to more than 80% of the non-infected classes.

Figure 3 Panel (b) shows the proportion of BTI ($S_U$ and $S_P$) and non-BTI ($S_{S}$) by different SARS-CoV-2 variants. The proportion of BTI with Delta increased until the SGR phase and thereafter, the proportion of BTI with Omicron began to rise. Assuming that the prediction scenario based on data was continued until November 30, 2022, approximately 85% of infections were BTI, and almost one-seventh of individuals infected with Omicron were non-vaccinated.

### Prevalence ratio of non-vaccinated to vaccinated groups

**Figure 4:**
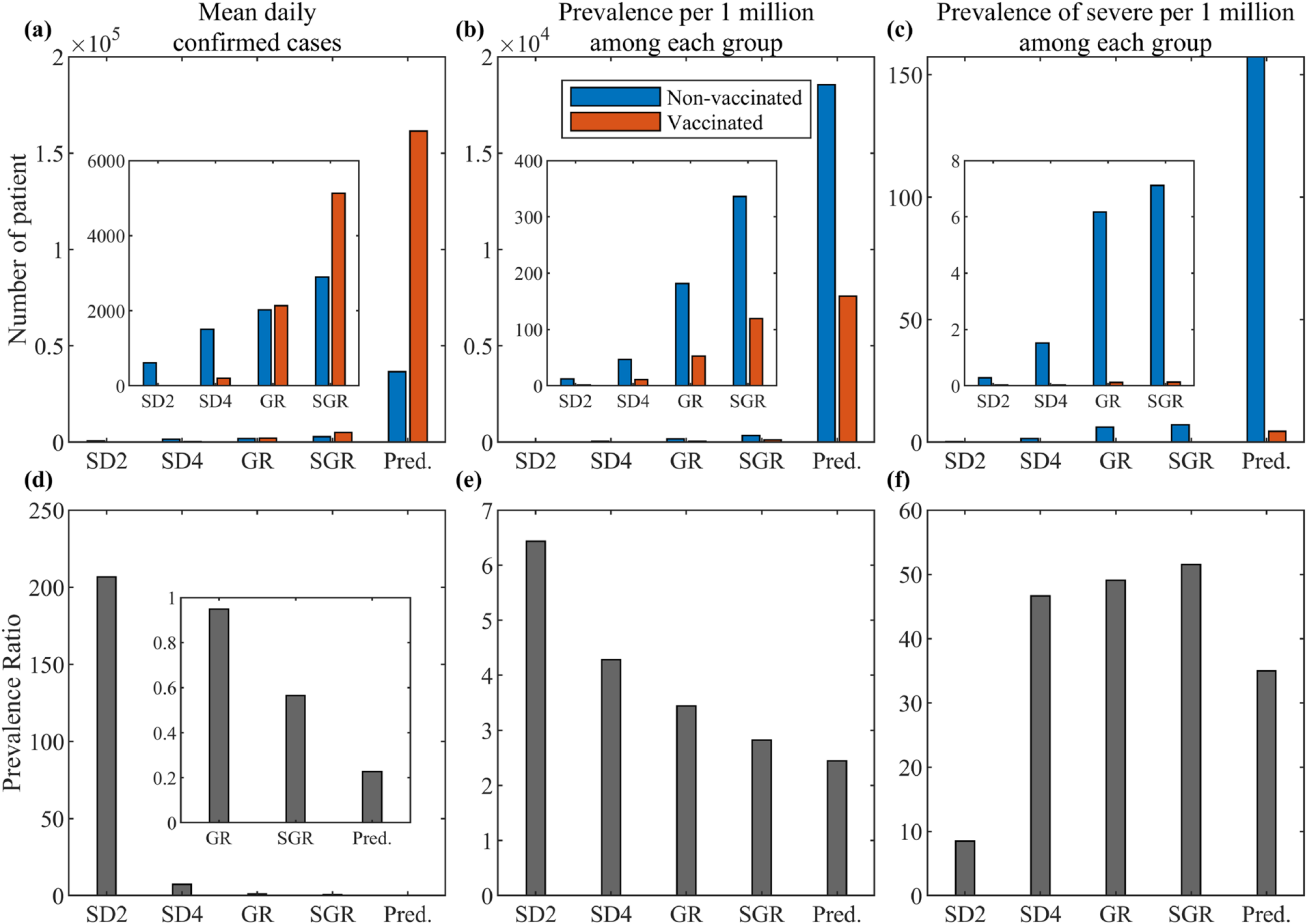
Prevalence of COVID-19 in non-vaccinated and vaccinated classes during the different SD phases and the prediction period. The red, blue, and gray bars show the prevalence of the vaccinated, non-vaccinated, and the prevalence ratio of the non-vaccinated to the vaccinated classes, respectively. The estimation period is from February 26, 2021 to February 2, 2022, and the prediction period is from February 3, 2022 to November 30, 2022. Panel (**a**) shows the mean daily confirmed cases, (**b,c**) prevalence of a case and severe case per one million, and (**d–f**) prevalence ratio calculated as (prevalence of non-vaccinated)/(prevalence of vaccinated). The insets show a magnification of the less visible bar graphs.

Figure 4 panel (a) shows that during SD2 and SD4, most of the confirmed cases are from the non-vaccinated classes, while in the SGR and prediction phases, most are from the vaccinated classes. Panels (b) and (c) show that there are more infections and severe cases in the non-vaccinated relative to the vaccinated. Moreover, until SGR, we can see that the prevalence of cases only reached around 350 and severe cases remained below 10. After February 2022, the prevalence of cases and severe cases increased to more than 18,000 and 150, respectively, in the non-vaccinated classes. Panel (d) shows that the prevalence of the non-vaccinated to the vaccinated among the confirmed cases during SD2 was around 200. In the prediction phase, the prevalence was only about 0.2. Panel (e) shows that the prevalence ratio of cases in the non-vaccinated to the vaccinated classes during SD2 is 6.4 and declined to 2.5 in the prediction period. Panel (f) shows that the prevalence ratio of severe cases during SD2 is less than 10, while during SD4, GR, and SGR, prevalence ratio of severe cases is around 50. In the prediction period, this reduced to 35.

### Effect of additional vaccines on the timing and peak of the Omicron wave

**Figure 5:**
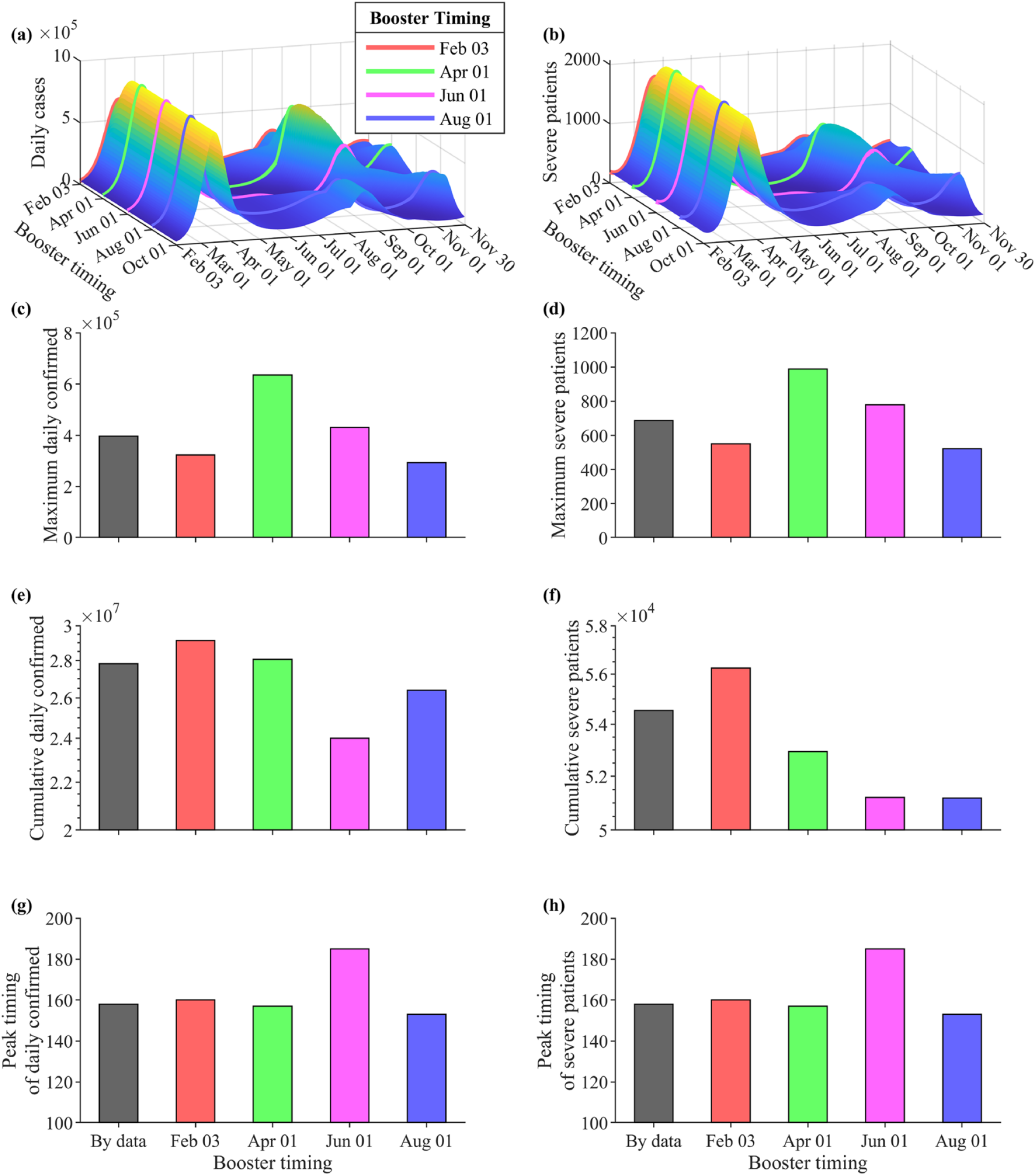
Simulation results when the additional 14 million vaccines were given at different times in 2022. The red, green, magenta, or blue curves and bars correspond to the results when the booster shots were given for a month starting on February 3, April 1, June 1, or August 1, 2022, respectively. Panels (**a,b**) show the daily cases and severe patients for each scenario. The black bars represent the simulation results using the vaccination data in Korea. Panels (**c–h**) illustrate the peak, cumulative, and timing of the second Omicron wave, respectively.

Figure 5 panels (a) and (b) show the model prediction results when 14 million vaccines were administered for 1 month starting on different dates in 2022. The red, green, magenta, and blue curves correspond to the scenario when the additional booster shots were administered on February 3, April 1, June 1, and August 1, 2022, respectively. In the simulations, we assume the value of $\mu$, as discussed in the “Materials and methods” section, based on the vaccination and severe patients data during this period. In all scenarios, the first Omicron peak was in mid-March. The maximum number of confirmed cases and severe patients were around 900,000 and 2000, respectively. However, the second Omicron peaks occurred at different times between August and September 2022 since the booster vaccination period varied. In panels (c) and (d), the maximum values of the peak in the second wave are much smaller than the first wave. Panels (e) and (f) show that proper vaccination timing can reduce the cumulative daily confirmed and severe patients up to four million and two thousand when the booster timing is on June 2022. The peaks of the second Omicron waves, as shown in panels (g) and (h), occur around the same day (day 153–160), except when the booster shots were administered for a month starting on June 1. In this case, the peak was delayed to day 185.

### Impact of a new variant to the daily incidence and severe patients

**Figure 6:**
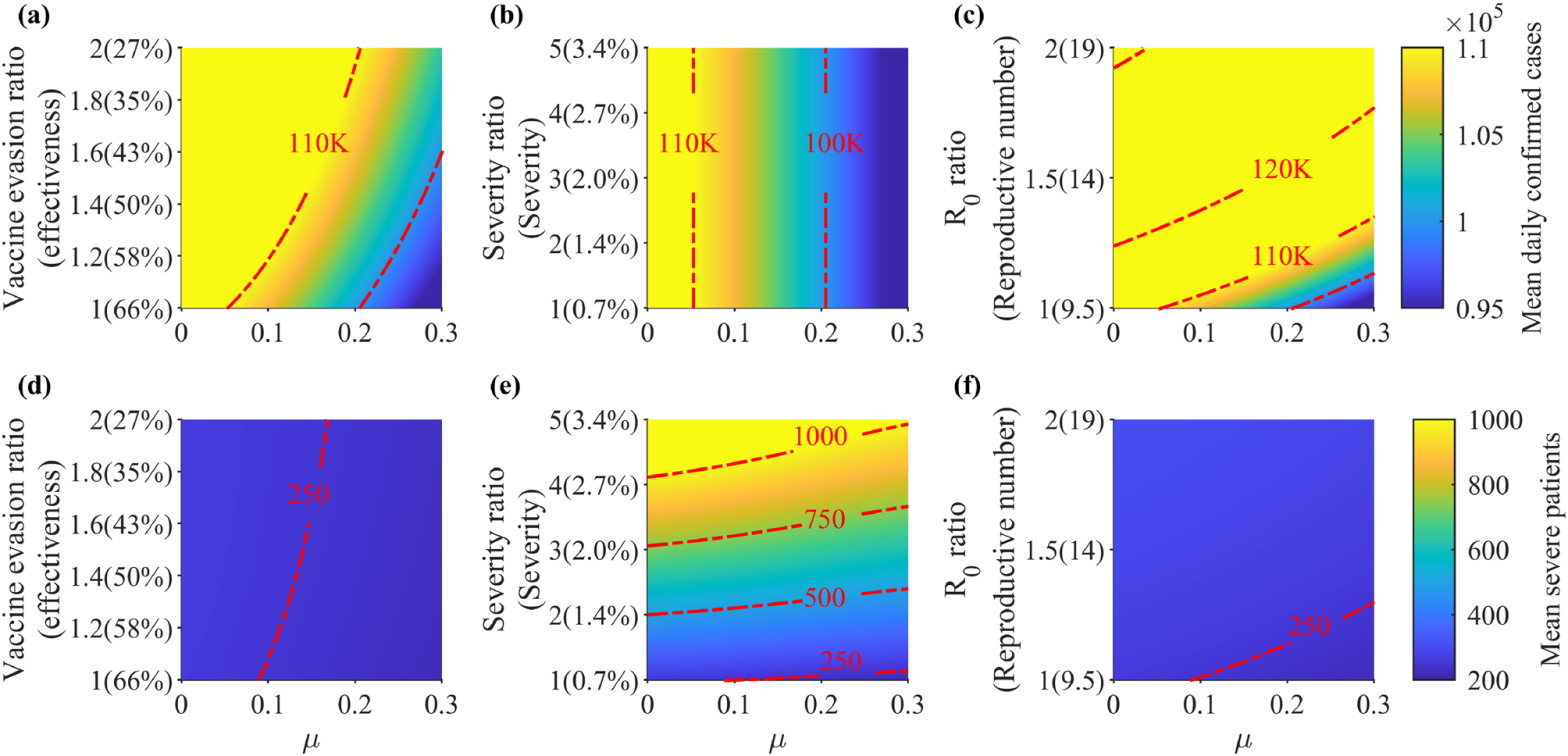
Simulation results characterizing new variants given varying vaccine evasiveness, disease severity, and ${\mathscr {R}}_0$ from December 2022 to June 2023 and $\mu \in (0,0.3)$. Vaccine evasion ratio of 1 means that the vaccine effectiveness against the variant is the same as the original Omicron variant, while a ratio of 2 means that vaccines are 27% effective against the new variant. The severity and ${\mathscr {R}}_0$ ratios indicate the ratio of severity rates and transmissibility of the new variant compared to the original Omicron variant. Panels (**a–c**) show the mean daily incidence, and panels (**d–f**) show the mean severe patients. The red dotted curves indicate the contours of the heatmap.

In Fig. 6, we investigate the effect of an emerging variant to the number of cases and severe patients assuming different characteristics such as vaccine evasion, severity, and transmissibility. The simulation period covers the end of the prediction phase until June 2023. Panels (a) to (c) show the mean daily confirmed cases and panels (d) to (f) show the mean number of severe patients from December 2022 to June 2023. If $\mu =0$, the mean daily confirmed cases is between 110,000 and 130,000. The maximum is observed in panel (c) when ${\mathscr {R}}_0$ is doubled. With the same properties as Omicron but with $\mu =0.3$, the mean daily confirmed cases is less than 100,000. If there are no changes in the severity of the new variant, then the mean number of severe patients is expected to be less than 500. The mean number of severe patients becomes over a thousand if the severity of the new variant is the same as that of Delta even with $\mu =0.3$.

## Discussion

We divided the simulation period into three: estimation period (February 26, 2021–February 3, 2022), prediction period (February 3, 2022–November 30, 2022), and projection period (November 30, 2022–June 30, 2023). In the estimation period, we obtained the values of the SD-related parameter $\mu$ by fitting the model to the cumulative confirmed cases data. In the prediction period, we assumed the $\mu$ value based on the recovery policy and severe cases since the confirmed cases are over the PCR testing capacity. We investigated the waning immunity of the population, prevalence ratio, and did scenario-based simulations. In the projection period, we assumed there is a new variant, possibly with different vaccine evasion, severity, and transmissibility properties compared to the existing variants, and varied the $\mu$ from 0 to 0.3.

Figure 1 captured the main events of the COVID-19 epidemic in Korea. The rise in the daily confirmed cases observed in July 2021 was influenced by the spread of the Delta variant, which has about twice the basic reproductive number compared to the pre-Delta variants. In response to the surge in cases, the Korean government implemented SD4, before the proportion of infections with the Delta variant reached 40%. Since the difference in $(1-\mu )$ was reduced from 0.37 (SD2) to 0.27 (SD4), the transmissibility of the disease during SD4 was reduced by about 27% compared to during SD2. Despite SD4, the reproductive number during this period remained around 1 since the Delta variant is more transmissible than the pre-Delta variants, and the primary vaccines may have already waned. Meanwhile, the reproductive number increased to greater than one during the Korean Thanksgiving Day celebration and Halloween. On November 1, 2021, as the vaccination with primary doses reached over 74% of the population and booster vaccination began, Korea announced the most eased SD, the GR policy. The reproductive number during this phase remained more than one and the daily confirmed cases reached over 10,000. Then the Omicron variant emerged and SGR policy was implemented. Here, the $\mu$ value was similar to the value of $\mu$ in SD2. The Omicron epidemic trend differed from that of Delta because the variants have different transmissibility and vaccine evasion properties.

From February 26, 2021 to February 3, 2022, there were three surges of COVID-19 infections: at the end of SD2, GR, and SGR. The surge in SD2 and SGR can be explained by the emergence of a more transmissible variant and by vaccine evasion. For the surge in GR, we investigated the immunity state of the population. Figure 3 shows the proportion of the population in the non-infected and infected classes. We observed that the waned population increased to 20% during GR phase, which made the non-infected population more susceptible to the disease. A similar phenomenon occurred between July and September 2022, which caused the second Omicron wave. Since early 2022, the recovered population had increased to 80%. For the model, this meant that most of the population do not infect other classes, there is a reduced population that can be infected, and thus, the peak of the next wave is reduced. Meanwhile, breakthrough infections increased to more than 80% during the Omicron wave, unlike in the Delta and pre-Delta periods.

In panels (a) and (d) of Fig. 4, the mean daily confirmed cases among the vaccinated is about five times more than the non-vaccinated during the prediction phase. This gap is reversed during SD2, where prevalence was six times greater in the non-vaccinated compared to the vaccinated. Meanwhile, the number of severe patients is directly related to the medical capacity. In panel (f), the prevalence of severe patients in the SGR period is 50 times more in the non-vaccinated than in the vaccinated. These results illustrate that although the number of cases among the vaccinated is much larger than the non-vaccinated, vaccines effectively prevent infection and severe symptoms. At the end of the SGR period, Korea approved the use of an antiviral drug for COVID-19. Hence, in the prediction period, we observe that the prevalence ratio reduced to 30. The question we now ask is, when is the most favorable timing to give the vaccines to the population?

Figure 5 depicts the projected daily cases and severe patients under different timing of administration of booster vaccines. All scenarios show a second Omicron wave between August and November 2022. The gray bars represent the data. The main objective of finding the appropriate booster timing is to delay the peak or reduce its size. Booster administration between April and June results to a high peak size in severe patients as shown in panel (d). Giving booster shots after September is inappropriate since the second omicron peak already occurred before September.

The smallest peak size in daily confirmed cases and severe patients is obtained when the booster shots are administered starting on August 1, 2022 (blue). However, under this scenario, the peak of the second Omicron wave is expected to occur 153 days after the first Omicron peak. The peak can be delayed the most when booster administration is started on June 1, 2022 (magenta), which is two months before the next epidemic wave is expected to occur assuming that no booster shots were given to the population. This scenario has the lowest cumulative confirmed cases and the lowest cumulative severe patients, but the peak size could reach 430,000. Considering the size and date of occurrence of the second Omicron peak, the most favorable time to administer the booster shots is between June and August 2022. These two scenarios have lower cumulative daily cases and severe patients compared to the data (gray). This strategy supports the vaccination plan of Denmark in 2021^29^.

The main reason for the surge in infections is the emergence of new variants with high transmissibility or vaccine evasion. Figure 6 illustrates scenarios considering a new variant that can have different properties, as in the case of 1H-2023 variant of Omicron. Without any variants and NPIs, Korea suffered about 110,000 cases per day and 300 severe patients in a day when 1H-2023 emerged. Transmissibility-related parameters cannot increase the severe cases up to 500 without NPIs. The severity-related parameter increases the number of severe patients by more than a thousand, even with the NPIs. This means that strategies which can reduce severity, such as the use of antiviral drugs or vaccines targeting the specific variant, are more important than maintaining NPIs.

There are several limitations to this study. Since we did not consider any inhomogeneity like regional or population structure, we cannot realize the differences in contact and characteristics of each group. So our study does not consider age-related differences in disease severity and prioritization in vaccination. Nevertheless, this study investigates the infection dynamics by various vaccines and variants. With the results, we emphasize the impact of vaccination, the importance of vaccination timing, and the strategies to minimize the impact of future variants. Unreported cases are another limitation of this research since during the first Omicron epidemic, the number of confirmed cases was greater compared to the daily PCR testing. For this reason, we used the severe data from the February 3, 2022 and divided the estimation and prediction periods.

Our future studies will be about intervention strategies considering heterogeneity in the population or region. We also plan to include unreported cases and study their effect in the epidemic situation. In formulating $\mu$, we assumed that the parameter values change every 2 weeks. One can explore using $\mu$ values on a weekly interval. However, this will double the number of unknown parameters and significantly increase the computational cost. This may require the use of different optimization algorithms (like metaheuristic methods) since local search methods (using the Matlab built-in function lsqcurvefit) may not work for high-dimensional problems. We also plan to develop a dashboard based on a mathematical model which differentiates between those who had COVID-19 and those who had not. In the dashboard, users can change the properties of the new variants and observe what may happen in the future. It can be useful for citizens, governments, and policymakers.

## Conclusion

In this research, we propose a mathematical model considering NPIs and PIs. Results suggest that vaccination is beneficial for citizens to protect themselves from severe symptoms of COVID-19. Moreover, vaccination campaigns should be implemented just before the expected surge in cases driven by the waning immunity of the population. Policymakers are advised to use mathematical modeling or other predictive methods in monitoring the waning of immunity. To address the potential threat of new variants, reducing the severity rate of the variants is a critical measure to prevent overwhelming the medical system.

## Materials and methods

### Data

From February 26, 2021 to December 31, 2022, we compiled the data on confirmed cases, severe cases, primary vaccinations, and booster vaccinations^8^. In the parameter estimation period (February 26, 2021 to February 3, 2022), we utilized the data on confirmed cases, primary and booster vaccinations. For the prediction period (February 3, 2022 to December 31, 2022), we used the primary and booster vaccination data. Severe patients data were used to compare with the simulation results of the model. To calculate the effectiveness of the vaccines administered in Korea, we considered the proportion of the population who were administered with a certain vaccine type^30^. The data on the proportion of Delta and Omicron variants were obtained from Korea Disease Control and Prevention Agency (KDCA)^31,32^ and compared with the model simulations as shown in Fig. 1.

**Figure 7:**
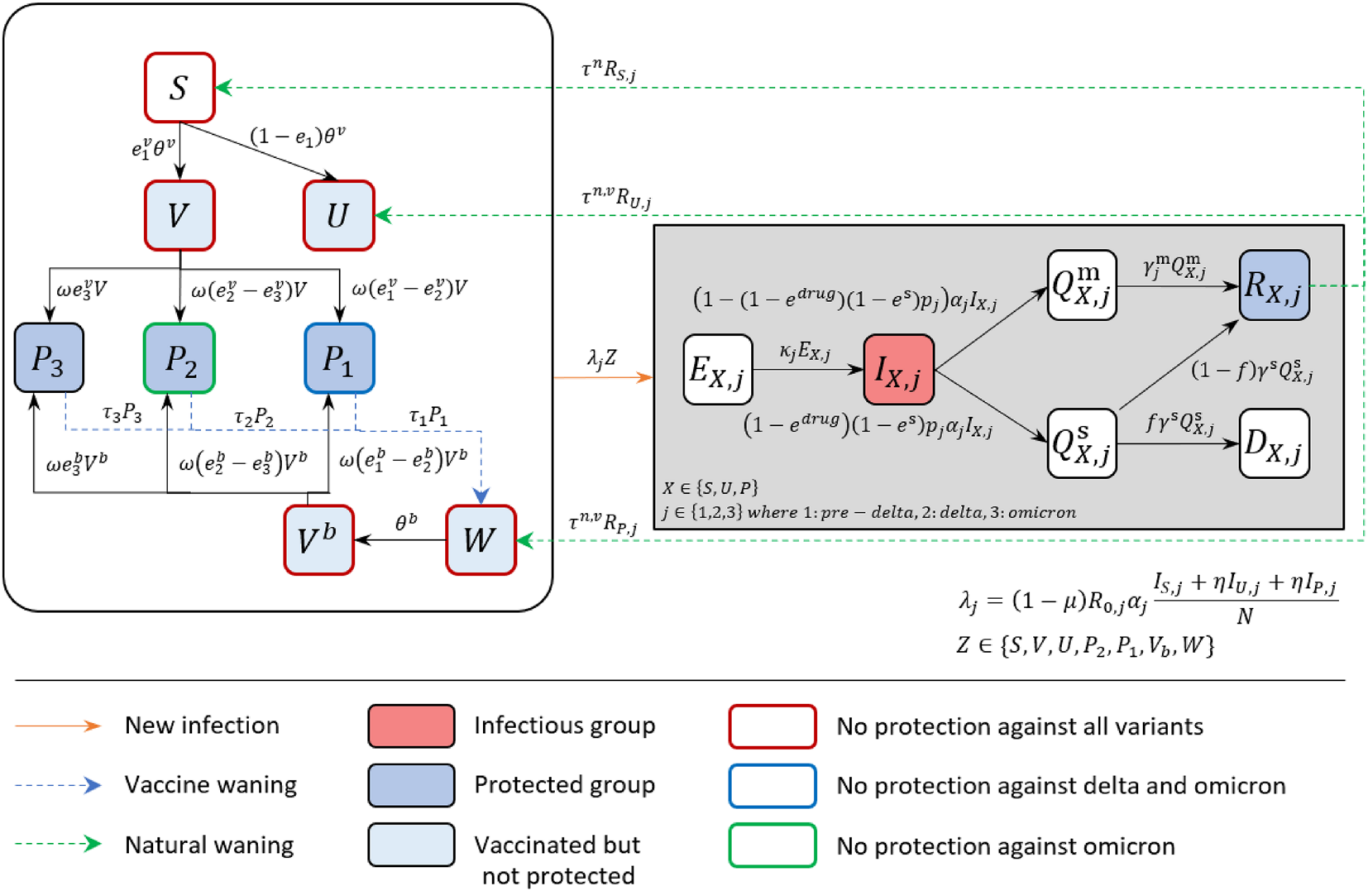
The model flowchart depicts the transmission of COVID-19 considering different variants, vaccinations, waning, and booster shots. The large round box group the compartments of non-infected people with different vaccine statuses. The compartments enclosed by red, green, and blue round boxes indicate individuals who can be infected by any variant, only Delta and Omicron, and only Omicron, respectively. The $P_j$ compartments represent the vaccinated individuals who are protected from the variants. The compartments inside the gray box denote infected individuals who are in one of several stages of infection, such as latent, infectious, isolated, recovered, or deceased.

### Mathematical model

We use a deterministic SEIQR model to describe the transmission of COVID-19 considering the variants, vaccination, waning, and booster shots. The model diagram in Fig. 7 shows that once susceptible individuals (*S*) are vaccinated, they are categorized into effectively (*V*) or ineffectively (*U*) vaccinated groups. The parameter $\theta ^v$ denotes the number of primary doses of vaccines administered daily, according to available data. The parameter $e^v_j$ represents the adjusted effectiveness of primary vaccines. Here, $j\in \{1,2,3\}$ denote infection with pre-Delta, Delta, and Omicron, respectively. We assume that individuals in *U* cannot get immunity against infection but have immunity against getting severe from vaccines. After an average of $1/\omega$ days from the primary vaccination, individuals in *V* become protected ($P_j$) against pre-Delta, Delta, or Omicron variants. We assume that individuals protected from Omicron are also protected from the other variants, and individuals protected from Delta are also protected from the pre-Delta variants. For example, individuals in $P_1$ may be infected by Delta or Omicron variants. As the immunity provided by vaccines wanes over time, the protected individuals ($P_j$) move to the waned *W* at a rate $\tau ^v_j$. When individuals in *W* receive booster shots, they move to the $V^b$ compartment and become protected again after an average of $1/\omega$ days. Similarly, according to the data, $\theta ^b$ represents the number of daily administered booster shots. The parameters $e^b_{j}$ denote the adjusted effectiveness of booster doses. Vaccine-induced immunity is assumed to wane exponentially. The calculated values of $e^v_{j}$, $e^b_{j}$, $\tau ^v_{j}$, and *f* are shown in Table 1 and the details of the calculation are given in Supplementary C.

Individuals in the susceptible and vaccinated compartments may be exposed ($E^X_j$) to any variants with forces of infection $\lambda _j(t)$. Here, $X\in \{S,U,P\}$ where *S*; *U*; and *P* are infected from *S*, *V*; *U*; $P_2$, $P_1$, $V^b$, and *W*, respectively. The parameter $\lambda _j (t)$ is defined as follows:

1 $$\begin{aligned} \lambda _j (t)=(1-\mu (t))R_{0,j}\alpha _j\frac{\Sigma _{X} I_{X,j}(t)}{N}. \end{aligned}$$

The expression $(1-\mu )$ in $\lambda _j(t)$ accounts for the reduction in transmission induced by NPIs such as social distancing. The basic reproduction numbers of pre-Delta, Delta, and Omicron are denoted as ${\mathscr {R}}_{0,1}, {\mathscr {R}}_{0,2}$, and ${\mathscr {R}}_{0,3}$, respectively. For brevity, we omit (*t*) notation on each compartment and parameter. All the compartments are time-dependent, and $\theta ^v$, $\theta ^b$, $\lambda _j$, $\mu$ are time-dependent parameters. Exposed individuals become infectious ($I_{X,j}$) after $1/\kappa _j$ days. Once they were identified by contract tracing or symptom onset, it will take about $1/\alpha _j$ days to be confirmed and distinguished as mild quarantined ($Q^{\text {m}}_{X,j}$) or severe quarantined ($Q^{\text {s}}_{X,j}$) cases according to severe rate ($p_j$). Severity can be reduced by vaccines ($e^{\text {s}}$) and antiviral drugs ($e^{drug}$), which are multiplied to the severity rate. An isolated individual either recovers ($R_{X,j}$) after $1/\gamma _j^{\text {m}}$ or $1/\gamma ^{\text {s}}$ days on average, or dies ($D_{X,j}$) from the disease with fatality rate *f*. Individuals who recover from the disease develop natural immunity against COVID-19 until their immunity wanes. In this study, we set the natural recovery waning period for the non-vaccinated ($1/\tau ^n$) as 350 days and for the vaccinated ($1/\tau ^{n,v}$) as 480 days^5,7^. The model parameters are listed in Table 1 and the model equations are in Supplementary B.

**Table 1:** The symbols and values used in the mathematical model.

Symbol	Description (units)	pre-$\delta$	$\delta$	o	Ref.
${\mathscr {R}}_{0,j}$	Basic reproduction number	2.87	5.08	9.5	^2,33,34^
$1/\kappa _j$	Mean latent period (days)	4	2	2	^35,36^
$1/\alpha$	Mean infectious period (days)	6	4	4	^36,37^
$p_j$	Proportion of severe cases	2.28%	3.39%	$0.68\%{*}$	^4,8,38^
*f*	Mean fatality rate among severe cases	$60.7\%^{\dagger }$	$60.7\%^{\dagger }$	$60.7\%^{\dagger }$	^8^
$1/\gamma ^\text {m}$	Mean duration of hospitalization for mild cases (days)	11.7	11.7	7	^39,40^
$1/\gamma ^\text {s}$	Mean duration of hospitalization for severe cases (days)	$11^{*}$	$11{*}$	$11{*}$	^40^
$e^v_j$	Adjusted effectiveness of primary vaccines	$91\%^{\dagger }$	$90\%^{\dagger }$	$63\%^{\dagger }$	^3,6,30^
$e^b_{j}$	Adjusted effectiveness of booster vaccines	–	$95\%^{\dagger }$	$66\%^{\dagger }$	^3,6,30^
$e^\text {s}$	Vaccine effectiveness against severe infections	$97\%$	$93\%$	$93\%$	^3,6^
$1/\tau ^n$	Mean waning rate of infection-induced immunity for non-vaccinated (days)	350	350	350	^7^
$1/\tau ^{n,v}$	Mean waning rate of infection-induced immunity for vaccinated (days)	480	480	480	^5^
$1/\tau ^{v}_j$	Mean waning rate of vaccine-induced immunity (days)	$349^{\dagger }$	$349^{\dagger }$	$138^{\dagger }$	^3,6,30^
$1/\omega$	Mean duration to have immune after vaccines (days)	14	14	14	^3,6^
$1/e^{drug}$	Effectiveness of antiviral against severity	89%	89%	89%	^41^

The subscripts $j=1,2,3$ refer to infection with pre-Delta, Delta, and Omicron variants, respectively.

*Assumed value.

$$\dagger$$Calculated value).

### Parameter estimation

The time-dependent parameter $\mu (t)$ is fitted by minimizing the square error of the cumulative number of cases using the model $\displaystyle \int \sum \limits _{j=1}^3 \alpha _j(I_{S,j} + I_{U,j} + I_{P,j})$ and the data from February 26, 2021 to February 3, 2022^8^. We assume that the value of $\mu (t)$ changes every two weeks. The least-squares formulation is solved using the MATLAB built-in function lsqcurvefit. The initial values of individuals exposed to Delta and Omicron variants when the first cases of each variant are confirmed are set to 1. We set the date of first confirmed case of the Delta and Omicron variants to April 27, 2021 and November 24, 2021, respectively^42–44^.

The effective reproductive number ${\mathscr {R}}(t)$ is calculated using next-generation method^45,46^. We obtain

2 $$\begin{aligned} {\mathscr {R}}(t)&={\mathscr {R}}_{0,1}(1-\mu (t))\frac{S+V+U+W+V_b}{N}\frac{I_{S,1}+ I_{U,1}+I_{P,1}}{\sum \limits _{j=1}^{3}(I_{S,j}+I_{U,j}+I_{P,j})} \nonumber \\&\quad +{\mathscr {R}}_{0,2}(1-\mu (t))\frac{S+V+U+W+V_b+P}{N}\frac{I_{S,2}+ I_{U,2}+I_{P,2}}{\sum \limits _{j=1}^{3}(I_{S,j}+I_{U,j}+I_{P,j})} \nonumber \\&\quad +{\mathscr {R}}_{0,3}(1-\mu (t))\frac{S+V+U+W+V_b+P+P_\delta }{N}\frac{I_{S,3}+ I_{U,3}+I_{P,3}}{\sum \limits _{j=1}^{3}(I_{S,j}+I_{U,j}+I_{P,j})}. \end{aligned}$$

### Bootstrapping

Because the estimation process has some uncertainty caused by oscillatory data or the estimation method, we performed an uncertainty analysis by resampling the cumulative confirmed data using a Poisson distribution for each data point. This process, called bootstrapping, is a method used for uncertainty analysis to obtain statistical information about the estimated parameters such as standard deviation or confidence interval^47^. The algorithm starts with the estimated model results from the data. We then apply perturbations to the model simulation results obtained from data fitting to obtain a certain number of simulated datasets. In this study, we re-estimated the parameters from 1000 different datasets that we generated using the Poisson error of each simulation point. As the model was fitted to the cumulative confirmed data, we generated cumulative confirmed datasets from the model simulation. Supplementary A shows the distribution of the re-estimated parameters. The mean value, standard deviation, and 95% confidence interval of each $\mu$ value are also given. Most of the confidence intervals are less than 1.0e−3.

### Modelling scenarios

We divided the simulation period into estimation, prediction, and projection periods. The modeling starts on February 26, 2021, the date when vaccination started. The estimation period includes the data-fitting process to quantify the social distancing level $\mu (t)$ and proportion of the variants. From February 3, 2022, we set the $\mu$ value based on the gradual easing of policies and used the vaccination data until November 30, 2022. The last estimated $\mu$ value was kept until March, then the $\mu$ value is set to 0.5 from April to July 2022, when the SD levels were lifted. The value of $\mu$ was decreased every month from August 2022 when outdoor mask writing was lifted. Figure 2 shows the model simulations under these assumptions on $\mu$.

Next, we investigate the proportion of the population of non-infected and infected people, and the prevalence of the disease in the non-vaccinated and vaccinated groups. Based on the model, the non-infected population can be divided into five groups: fully protected against all the variants ($P_3$), partially protected against the variants ($P_1,P_2$), vaccine waned population ($W,V^b$), ineffectively vaccinated (*U*), and unprotected (*S*, *V*). The infected groups are divided according to the variant and whether it is a BTI (subscript *U*, *P*) or non-BTI (subscript *S*). We look at the dynamics of the proportions of the non-infected and infected groups as more individuals got vaccinated with primary and booster doses, and as the variants emerged. Results shown in Fig. 5 uses the same assumption on SD as in the previous scenario, but a different assumption about vaccination. The simulation with different vaccination timing was performed to investigate the importance of timely vaccination.

The projection period is from November 30, 2022 to June 30, 2023. Here, we examine the impact of vaccine evasion, disease severity, and Omicron’s transmissibility relative to Delta. We are interested in the mean number of daily cases and severe patients under different assumptions on the value of $\mu$. We vary the values for vaccine evasion from $e^b_3$ to $e^b_3-(e^b_2-e^b_3)$. The range of severity is from $p_3$ to $p_2$. We assume that the reproductive number of the new variant could be twice that of the Omicron variant.

## Supplementary Information

Supplementary Information.

## Data Availability

The study uses the software MATLAB 2022b. All the data used in this study is available in the references^8,30–32^.
