# Supplementary material for: Modelling the effects of social distancing, antiviral therapy, and booster shots on mitigating Omicron spread
Source: Sci Rep. 2023 Apr 27;13:6914. doi: 10.1038/s41598-023-34121-y (PMC10139668; doi:10.1038/s41598-023-34121-y)
Supplement: Supplementary file 1 — Supplementary Information. [file 41598_2023_34121_MOESM1_ESM.pdf]

Supplementary

Jongmin Lee[1], Renier Mendoza[2], Victoria May P. Mendoza[2], Jacob Lee[3], Yubin Seo[3], Eunok Jung[1,*]

1 Department of Mathematics, Konkuk University, Seoul, 05029, South Korea
2 Institute of Mathematics, University of the Philippines Diliman, Quezon City, 1101, Philippines
3 Division of Infectious Disease, Department of Internal Medicine, Kangnam Sacred Heart Hospital, Hallym University College of medicine, Seoul, 07441, South Korea

* ***Correspondence:*** `junge@konkuk.ac.kr`

A Uncertainty analysis result

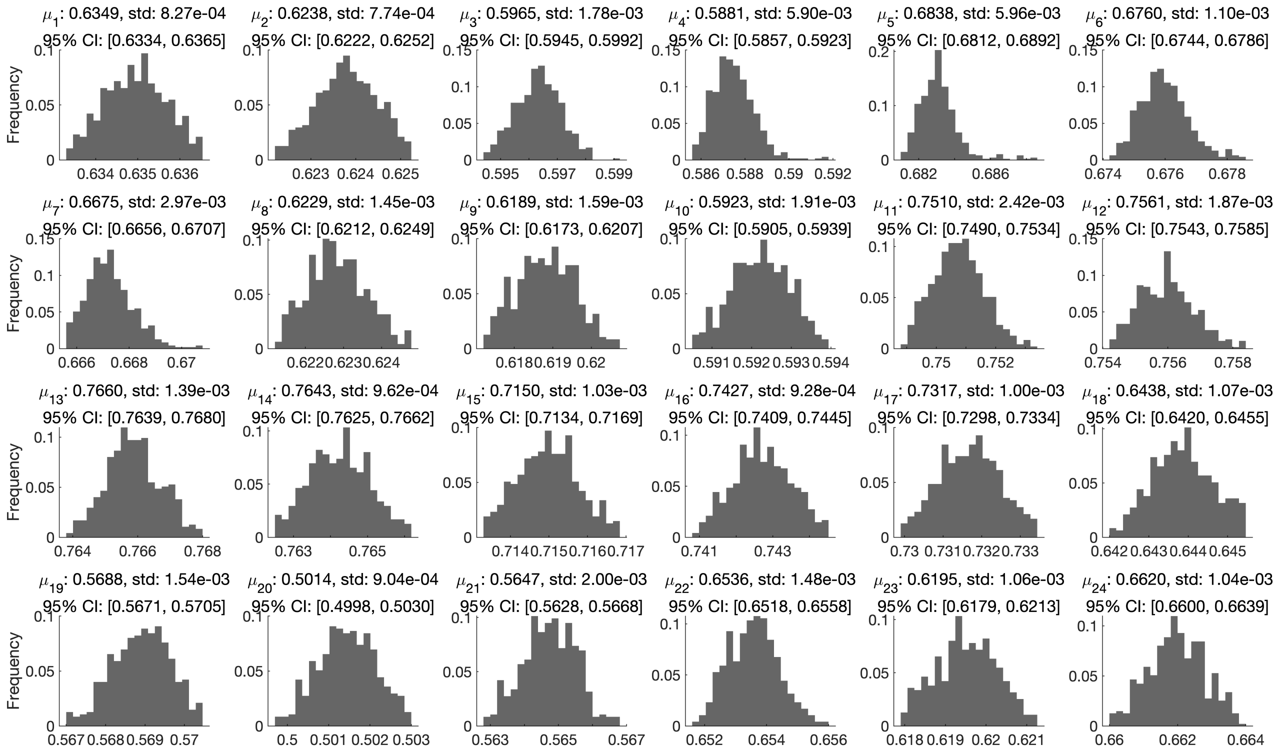

Figure 1. The bootstrap results as distribution of each $\mu(t)$ from one thousand times realization. The mean, standard deviation, 95% confidence interval from the distribution are shown.

Figure 1 shows the Bootstrap result with a thousand realization of estimated μ values. Each histogram indicates the distribution of estimated μ values from a thousand different datasets obtained by the Poisson error of the simulation result. Details of the statistical information from the distributions are in Table 1.

Bootstrapping gives us statistical information about the estimated parameter. Table 1 shows the mean, median, mode, standard deviation, 95% confidence interval, and estimated value from the data with the 1000 realization. The

NPIs	mean μ during NPIs	μ with two-week intervals	estimated	mean	median	mode	SD	CI
SD2	0.6301	1	0.6350	0.631917	0.631881	0.631603	0.000192	[0.63165, 0.63233]
		2	0.6239	0.620035	0.620038	0.619426	5.92E-05	[0.61992, 0.62012]
		3	0.5963	0.599836	0.599826	0.599348	7.26E-05	[0.59972, 0.59999]
		4	0.5873	0.589804	0.58981	0.589524	7.49E-05	[0.58969, 0.58993]
		5	0.6829	0.679992	0.679968	0.679767	0.000104	[0.67989, 0.68012]
		6	0.6758	0.680387	0.680364	0.680059	0.000274	[0.68017, 0.68068]
		7	0.6671	0.669645	0.669631	0.669405	0.000356	[0.66956, 0.66974]
		8	0.6228	0.620041	0.620034	0.619803	0.000121	[0.61994, 0.62013]
		9	0.6188	0.619985	0.619977	0.619688	0.000305	[0.6199, 0.62005]
		10	0.5922	0.589689	0.589682	0.589516	0.000151	[0.58959, 0.58976]
SD4	0.7327	11	0.7505	0.750355	0.750338	0.749844	0.000288	[0.75016, 0.75056]
		12	0.7559	0.760158	0.760159	0.75968	0.000273	[0.75987, 0.76031]
		13	0.7658	0.770176	0.770171	0.76971	5.32E-05	[0.77011, 0.77026]
		14	0.7642	0.760267	0.760255	0.759822	0.000364	[0.76016, 0.76034]
		15	0.7149	0.710189	0.71018	0.709907	0.000245	[0.71011, 0.71028]
		16	0.7427	0.739975	0.739988	0.736746	0.000115	[0.73986, 0.74007]
		17	0.7317	0.730061	0.730047	0.729537	0.000368	[0.72996, 0.73012]
		18	0.6438	0.640103	0.640089	0.639844	0.000129	[0.64, 0.64023]
GR	0.5433	19	0.5690	0.569852	0.569872	0.569302	0.000163	[0.56947, 0.57001]
		20	0.5015	0.499897	0.499906	0.499381	0.000132	[0.49963, 0.50005]
		21	0.5647	0.560149	0.56017	0.559684	0.000118	[0.5599, 0.56031]
SGR	0.6435	22	0.6536	0.65012	0.650134	0.649592	0.000112	[0.64987, 0.6503]
		23	0.6195	0.62014	0.62014	0.619753	9.25E-05	[0.61997, 0.62029]
		24	0.6619	0.660226	0.660227	0.659798	7.72E-05	[0.66013, 0.66037]

Table 1. The estimated μ value for each phase of NPIs and time division. Statistical information for each μ value is given in the form of mean, median, mode, standard deviation (SD), and confidence interval (CI). These parameters are induced by $lsqcurvefit$ function in MATLAB and the cumulative confirmed data from February 26, 2021 to February 3, 2022.

difference between the estimated value from the data and the mean value of all realization is lesser than 0.005, and standard deviations are lesser than 0.0005 for all μ. During the SD2, SD4, GR, and SGR, the ranges of $\mu(t)$ are (0.59,0.68), (0.64, 0.77), (0.49, 0.57), and (0.62, 0.66), respectively. Based on the estimation, the most eased phase is GR ($\mu = 0.54$), and the most strict phase is SD4 ($\mu = 0.73$).

B Model equations

Our model is an SEIQRD compartment model considering the severity of quarantined individuals. Each infection-related compartment (E, I, Q^{m}, Q^{s}, R, D) is categorized according to vaccination state and variant, which are distinguished by subscripts. The subscript j represents the type of variant. $j = 1$, 2, or 3 refers to infection with pre-Delta, Delta, or Omicron, respectively. The Subscript X represents the immune state of infected individuals. $X = S$; U; or P refers to infected individuals from S, V; U; P_1, P_2, P_3, W, V^b, respectively. Equations 1 to 6 describe the infection-related states.

$$\begin{cases} \frac{dE_{S,j}}{dt} &= (S+V)\lambda_1 - \kappa_j E_{S,j} \\ \frac{dE_{U,j}}{dt} &= (U)\lambda_2 - \kappa_j E_{U,j} \\ \frac{dE_{P,1}}{dt} &= (W+V^b)\lambda_3 - \kappa_1 E_{P,1} \\ \frac{dE_{P,2}}{dt} &= (W+V^b+P_1)\lambda_3 - \kappa_2 E_{P,2} \\ \frac{dE_{P,3}}{dt} &= (W+V^b+P_1+P_2)\lambda_3 - \kappa_3 E_{P,3} \end{cases} \tag{1}$$

$$\begin{cases} \frac{dI_{S,j}}{dt} &= \kappa_j E_{S,j} - \alpha_j I_{S,j} \\ \frac{dI_{U,j}}{dt} &= \kappa_j E_{U,j} - \alpha_j I_{U,j} \\ \frac{dI_{P,j}}{dt} &= \kappa_j E_{P,j} - \alpha_j I_{P,j} \end{cases} \tag{2}$$

$$\begin{cases} \frac{dQ^{\mathrm{m}}_{S,j}}{dt} &= (1-(1-e^{drug})p_j)\alpha_j I_{S,j} - \gamma^{\mathrm{m}}_j Q^{\mathrm{m}}_{S,j} \\ \frac{dQ^{\mathrm{m}}_{U,j}}{dt} &= (1-(1-e^{drug})(1-e^{\mathrm{s}})p_j)\alpha_j I_{U,j} - \gamma^{\mathrm{m}}_j Q^{\mathrm{m}}_{U,j} \\ \frac{dQ^{\mathrm{m}}_{P,j}}{dt} &= (1-(1-e^{drug})(1-e^{\mathrm{s}})p_j)\alpha_j I_{P,j} - \gamma^{\mathrm{m}}_j Q^{\mathrm{m}}_{P,j} \end{cases} \tag{3}$$

$$\begin{cases} \frac{dQ^{\mathrm{s}}_{S,j}}{dt} &= (1-e^{drug})p_j\alpha_j I_{S,j} - \gamma^{s} Q^{\mathrm{s}}_{S,j} \\ \frac{dQ^{\mathrm{s}}_{U,j}}{dt} &= (1-e^{drug})(1-e^{\mathrm{s}})p_j\alpha_j I_{U,j} - \gamma^{s} Q^{\mathrm{s}}_{U,j} \\ \frac{dQ^{\mathrm{s}}_{P,j}}{dt} &= (1-e^{drug})(1-e^{\mathrm{s}})p_j\alpha_j I_{P,j} - \gamma^{s} Q^{\mathrm{s}}_{P,j} \end{cases} \tag{4}$$

$$\begin{cases} \frac{dR_{S,j}}{dt} &= \gamma^{\mathrm{m}}_j Q^{\mathrm{m}}_{S,j} + (1-f)\gamma^{\mathrm{s}} Q^{\mathrm{s}}_{S,j} - \tau^{n} R_{S,j} \\ \frac{dR_{U,j}}{dt} &= \gamma^{\mathrm{m}}_j Q^{\mathrm{m}}_{U,j} + (1-f)\gamma^{\mathrm{s}} Q^{\mathrm{s}}_{U,j} - \tau^{n,v} R_{U,j} \\ \frac{dR_{P,j}}{dt} &= \gamma^{\mathrm{m}}_j Q^{\mathrm{m}}_{P,j} + (1-f)\gamma^{\mathrm{s}} Q^{\mathrm{s}}_{P,j} - \tau^{n,v} R_{P,j} \end{cases} \tag{5}$$

$$\begin{cases} \frac{dD_{S,j}}{dt} &= f\gamma^{\mathrm{s}} Q^{\mathrm{s}}_{S,j} \\ \frac{dD_{U,j}}{dt} &= f\gamma^{\mathrm{s}} Q^{\mathrm{s}}_{U,j} \\ \frac{dD_{P,j}}{dt} &= f\gamma^{\mathrm{s}} Q^{\mathrm{s}}_{P,j}. \end{cases} \tag{6}$$

The susceptible compartments are also categorized according to vaccination state. S and V are unprotected groups since they are not vaccinated or need more time to get immunity. The ineffectively vaccinated group (U) cannot get immunity by any vaccine. Any variant can infect the unprotected and ineffectively vaccinated groups. The effectiveness of vaccines against each variant is different, and hence, V may become P_1, P_2, or P_3, which means fully protected against some or all variants. As vaccine-induced immunity of individuals in P_j wane over time, they move to the W compartment. Individuals in W and U can get booster shots, but U cannot get immunity in our

assumption. Right after receiving booster shots, individuals in W move to V_b, and eventually to P_j.

$$\begin{cases} \frac{dS}{dt} = & -(\Sigma_{j=1}^{3}\lambda_j)S - \theta^v + \tau^n R_j \\ \frac{dV}{dt} = & -(\Sigma_{j=1}^{3}\lambda_j)V + e_1\theta^v - \omega V \\ \frac{dU}{dt} = & -(\Sigma_{j=1}^{3}\lambda_j)U + (1-e_1)\theta^v + \tau^{n,v} R_{U,j} \\ \frac{dP_1}{dt} = & -(\Sigma_{j=2}^{3}\lambda_j)P_1 + (e_1 - e_2)\omega V - \tau_1^v P_1 + (e_1^b - e_2^b)\omega V^b \\ \frac{dP_2}{dt} = & -\lambda_3 P_2 + (e_2 - e_3)\omega V - \tau_2^v P_2 + (e_2^b - e_3^b)\omega V^b \\ \frac{dP_3}{dt} = & e_3\omega V - \tau_3^v P_3 + e_3^b \omega V^b \\ \frac{dW}{dt} = & -(\Sigma_{j=1}^{3}\lambda_j)W + \tau_1^v P_1 + \tau_2^v P_2 + \tau_3^v P_3 - \theta^b + \tau^n R_{P,j} \\ \frac{dV^b}{dt} = & -(\Sigma_{j=1}^{3}\lambda_j)V^b + \theta^b - \omega V^b. \end{cases} \tag{7}$$

The forces of infection consider the variants and their transmissibility, social distancing, and transmission reduction by vaccines. The forces of infection are defined as

$$\begin{cases} \lambda_1 = & (1-\mu)\mathcal{R}_{0,1}\alpha_1 \frac{(I_{S,1}+I_{U,1}+I_{P,1})}{N} \\ \lambda_2 = & (1-\mu)\mathcal{R}_{0,2}\alpha_2 \frac{(I_{S,2}+I_{U,2}+I_{P,2})}{N} \\ \lambda_3 = & (1-\mu)\mathcal{R}_{0,3}\alpha_3 \frac{(I_{S,3}+I_{U,3}+I_{P,3})}{N}. \end{cases} \tag{8}$$

At the beginning of our estimation, vaccine, Delta, and Omicron-related compartments are zero because there are no vaccines and such variants. Since we have no data on infected people who are not yet confirmed, $E_{S,1}(0) = 400/\kappa_1 = 1600$ and $I_{S,1}(0) = 400/\alpha = 2400$ are calculated from average daily confirmed cases at the end of February 2021. The initial values for $Q_{S,1}^{\text{s}}, Q_{S,1}^{\text{m}}, R_{S,1}$, and $D_{S,1}$ are obtained from the data [1]. The initial value for S is calculated as the remaining number of individuals based on the total population and other initial values. With these, our proposed mathematical model consists of Equations 1-8 with initial values given in Table 2.

Symbol	Value	Description (units)	Ref.
N	51,705,905	Population of Korea in March 2021	[2]
$E_1(0)$	1,600	Initial value for unvaccinated latent group infected by pre-delta variant	Calculated
$I_1(0)$	2,400	Initial value for unvaccinated infectious group infected by pre-delta variant	Calculated
$Q_1^{\mathrm{m}}(0)$	7,313	Initial value for unvaccinated isolated group with mild symptom infected by pre-delta variant	[1]
$Q_1^{\mathrm{s}}(0)$	144	Initial value for unvaccinated isolated group with severe symptom infected by pre-delta variant	[1]
$R_1(0)$	79,880	Initial value for unvaccinated recovered group infected by pre-delta variant	[1]
$D_1(0)$	1,585	Initial value for unvaccinated death group infected by pre-delta variant	[1]
$S(0)$	51,612,983	Initial value of susceptible group	Calculated
E^{δ}_{import}	1	Initial expected patient when the first delta case was confirmed	Estimated
E^{o}_{import}	1	Initial expected patient when the first omicron case was confirmed	Estimated

Table 2. Initial value for model compartments; other initial of compartment are zero.

C Calculation of the parameters in our model

Vaccine-related model parameters (τ_j^v, e_j^v, e_j^b) are calculated based on the effectiveness at 2 and 20 weeks from the second dose for mRNA and viral-vector type after inoculation of booster shot. Two weeks after primary vaccination, the effectiveness of Pfizer against Delta (Omicron) is 91% (65%) and of Moderna against Delta (Omicron) is 95% (75%). It reduced to 67% (11%) and 76% (15%) twenty weeks after primary vaccination. Since the proportion of the population in Korea who were given mRNA as primary doses is 75.4% and who got Moderna vaccine is 19.6%, we can calculate the total effectiveness of primary vaccine at 2 and 20 weeks from the second does. For example, the effectiveness of mRNA vaccine against Delta is $0.804 \times 0.91 + 0.196 \times 0.95 = 0.92$, and the effectiveness of primary vaccine against Delta is $0.2467 \times 0.83 + 0.7543 \times 0.92 = 0.9$ Similarly, weighted arithmetic mean is used to calculate e_j^v, e_j^v, e_j^b from $e_j^{\mathrm{pre},v}$, $e_j^{\mathrm{pre},vw}$, $e_j^{\mathrm{pre},b}$, respectively. The values of the parameters e_j^{pre}, $e_{w,j}^{\mathrm{pre}}$, $e_{b,j}^{\mathrm{pre}}$, which we found from the literature, are stated in Table 3. Also, we assume that vaccine-induced immunity wanes exponentially at a rate τ_j^v. Then $h_{\mathrm{eff}}(t) := e_j^v e^{-\tau_j^v t}$. Since we know two values of vaccine effectiveness at two different time points, then

$$
\begin{aligned}
& h_{\mathrm{eff}}(126) = e_j^v e^{-126\tau_j^v} = e_j^{vw} \\
\implies \quad & \tau_j^v = -\log(e_j^{vw}/e_j^v)/126.
\end{aligned}
$$

At last, $f = 60.7\%$ is computed as the percentage of dead over cumulative severe cases. All calculation results are shown in Table 3.

Symbol	Description (units)		pre-δ	δ	o	Ref.
$e_j^{\text{pre,v}}$	Vaccine effectiveness against symptomatic disease after dose 2	viral-vector (2 weeks)	83%	83%	49%	[3, 4]
		mRNA (2 weeks)	95%	92%	67%	
$e_j^{\text{pre,vw}}$	Vaccine effectiveness against symptomatic disease after dose 2	viral-vector (20weeks)	76%	48%	4%	[3, 4]
		mRNA (20 weeks)	-%	69%	12%	
$e_j^{\text{pre},b}$	Vaccine effectiveness against symptomatic disease 2-4 weeks after booster shot	viral-vector (primary)	-%	95%	62%	[3, 4]
		mRNA (primary)	-%	95%	67%	
e_j^v	Adjusted effectiveness of mRNA and viral vector vaccines		91%	90%	63%	Calculated
e_j^{vw}	Adjusted effectiveness of mRNA and viral vector vaccines after 5 weeks		-%	64%	10%	Calculated
$1/\tau_j^v$	Mean waning duration of vaccine-induced immunity (day)		349	349	138	Calculated
e_j^b	Adjusted effectiveness of booster vaccines		-%	95%	66%	Calculated
$1/\omega$	Mean duration to have immune after normal vaccines (days)		14	14	14	[3, 4]

Table 3. The raw data for vaccine-related parameters. e_j^v, τ_j^v, e_j^b, and ω are used in the mathematical model.

References

1. Kdca press release (feb 26, 2021). `https://www.kdca.go.kr/board/board.es?mid=a20501010000&bid=0015&list_no=712549&cg_code=&act=view&nPage=99`. Accessed: March 8, 2023.
2. Korean statistical information service (population by administrative district). `https://kosis.kr/statisticsList/statisticsListIndex.do?publicationYN=Y&statId=2008001&outLink=Y&entrType=#content-group`. Accessed: March 8, 2023.
3. N. Andrews *et. al.* Duration of protection against mild and severe disease by covid-19 vaccines. *New England Journal of Medicine*, 386(4):340–350, 2022. PMID: 35021002.
4. N. Andrews *et. al.* Covid-19 vaccine effectiveness against the omicron (b.1.1.529) variant. *New England Journal of Medicine*, 386(16):1532–1546, 2022. PMID: 35249272.
